# Substrate Specificity and Inhibitor Sensitivity of Plant UDP-Sugar Producing Pyrophosphorylases

**DOI:** 10.3389/fpls.2017.01610

**Published:** 2017-09-20

**Authors:** Daniel Decker, Leszek A. Kleczkowski

**Affiliations:** Department of Plant Physiology, Umeå Plant Science Center, Umeå University Umeå, Sweden

**Keywords:** enzyme structure-function analyses, enzyme substrate specificity, nucleotide sugar synthesis, UDP-fructose, UDP-fucose, UDP-*N*-acetyl glucosamine pyrophosphorylase, UDP-sugar pyrophosphorylase, UDP-glucose pyrophosphorylase

## Abstract

UDP-sugars are essential precursors for glycosylation reactions producing cell wall polysaccharides, sucrose, glycoproteins, glycolipids, etc. Primary mechanisms of UDP sugar formation involve the action of at least three distinct pyrophosphorylases using UTP and sugar-1-P as substrates. Here, substrate specificities of barley and *Arabidopsis* (two isozymes) UDP-glucose pyrophosphorylases (UGPase), *Arabidopsis* UDP-sugar pyrophosphorylase (USPase) and *Arabidopsis* UDP-*N*-acetyl glucosamine pyrophosphorylase2 (UAGPase2) were investigated using a range of sugar-1-phosphates and nucleoside-triphosphates as substrates. Whereas all the enzymes preferentially used UTP as nucleotide donor, they differed in their specificity for sugar-1-P. UGPases had high activity with D-Glc-1-P, but could also react with Fru-1-P and Fru-2-P (*K*_m_ values over 10 mM). Contrary to an earlier report, their activity with Gal-1-P was extremely low. USPase reacted with a range of sugar-1-phosphates, including D-Glc-1-P, D-Gal-1-P, D-GalA-1-P (*K*_m_ of 1.3 mM), β-L-Ara-1-P and α-D-Fuc-1-P (*K*_m_ of 3.4 mM), but not β-L-Fuc-1-P. In contrast, UAGPase2 reacted only with D-GlcNAc-1-P, D-GalNAc-1-P (*K*_m_ of 1 mM) and, to some extent, D-Glc-1-P (*K*_m_ of 3.2 mM). Generally, different conformations/substituents at C2, C4, and C5 of the pyranose ring of a sugar were crucial determinants of substrate specificity of a given pyrophosphorylase. Homology models of UDP-sugar binding to UGPase, USPase and UAGPase2 revealed more common amino acids for UDP binding than for sugar binding, reflecting differences in substrate specificity of these proteins. UAGPase2 was inhibited by a salicylate derivative that was earlier shown to affect UGPase and USPase activities, consistent with a common structural architecture of the three pyrophosphorylases. The results are discussed with respect to the role of the pyrophosphorylases in sugar activation for glycosylated end-products.

## Introduction

UDP-sugar formation is an essential pre-requirement for any cell to produce larger and more complex carbohydrate-containing compounds. Production of UDP-sugars involves either primary mechanisms, where an UDP-sugar is produced from a sugar molecule that is activated by linking it with an UDP moiety, resulting in a more reactive compound (Kleczkowski and Decker, 2015), or secondary mechanisms, where an already produced UDP-sugar is converted to another one (Reiter, 2008; Yin et al., 2011). In plants, the primary mechanisms of UDP-sugar formation involve three UTP-dependent pyrophosphorylases, differing in specificity for sugar-1-P, which serves as second substrate. UDP-glucose pyrophosphorylase (UGPase) is considered to be more or less specific for glucose-1-P (Glc-1-P), and has by far the highest activity among the pyrophosphorylases (Kleczkowski et al., 2010; Kleczkowski and Decker, 2015). This is in contrast to UDP-sugar pyrophosphorylase (USPase), which uses a variety of sugar-1-phosphates as substrates (Kotake et al., 2004; Damerow et al., 2010; Kleczkowski et al., 2011a), and to UDP-*N*-acetylglucosamine pyrophosphorylase (UAGPase), which prefers *N*-acetylglucosamine-1-P (GlcNAc-1-P) and *N*-acetylgalactosamine-1-P (GalNAc-1-P) as substrates (Yang et al., 2010). All these enzymes are predominantly located in the cytosol (Kleczkowski et al., 2010; Kleczkowski and Decker, 2015), with the exception of a unique type of UGPase, which is entirely based in chloroplasts where it serves as an essential step in sulfolipid biosynthesis (Okazaki et al., 2009). As the synthesis of the UDP-sugars is a fully reversible reaction, each pyrophosphorylase can also be involved in the production of a given sugar-1-P from the respective UDP-sugar, and thus contributing to an equilibrium concentration between those metabolites.

Once made by the pyrophosphorylases, UDP-sugars then serve as substrates to a myriad of glycosyltransferase activities, which transfer the monosaccharide residue from a given UDP-sugar to an acceptor molecule. The acceptor could be another sugar (e.g., for sucrose or trehalose formation), a polysaccharide (e.g., cellulose, hemicellulose, pectin formation), a protein (glycoprotein formation), a lipid (glycolipid formation), and many others (Feingold and Avigad, 1980; Lairson et al., 2008; Yonekura-Sakakibara, 2009; Bar-Peled and O’Neill, 2011). A given UDP-sugar molecule can also be interconverted to another UDP-sugar by the action of specific epimerases and via other mechanisms, including – among others - a conversion of UDP-Glc to UDP-glucuronic acid (UDP-GlcA) by UDP-Glc dehydrogenase (Kleczkowski and Decker, 2015).

Extracts from higher plants have been shown to contain about 30 different types of nucleotide sugars, most of them UDP-sugars, and for some of them no known synthetic pathways have been demonstrated (Bar-Peled and O’Neill, 2011). Given the metabolic importance of UDP-sugars and the fact that they serve as precursors to most of biomass in nature (Kotake et al., 2010), surprisingly little is known about substrate specificity of the pyrophosphorylases catalyzing the primary reactions leading to glycosylated end-products, and even less about the relative contribution of each enzyme to these cellular pathways.

To contribute to that, in the present study, we measured the activities of several purified recombinant UDP-sugar producing pyrophosphorylases and compared their substrate specificities with respect to an array of sugar-1-P. The structure/function analyses of sugar-1-phosphates and their binding sites in the homology-derived structural models for the three pyrophosphorylases were compared with the substrate specificities that were experimentally determined for these enzymes. We also demonstrated that an inhibitor earlier shown to affect UGPase and USPase activities (Decker et al., 2017), had similar effects on purified UAGPase2. The data are discussed with respect to structural determinants of substrate binding and to possible roles of each of the pyrophosphorylases *in vivo.*

## Materials and Methods

### Recombinant UGPases, USPase and UAGPase2

Barley UGPase, *Arabidopsis* UGPase1 and UGPase2, and *Arabidopsis* USPase were heterologously expressed in *Escherichia coli* and purified to homogeneity as earlier reported (Martz et al., 2002; Meng et al., 2008; Decker et al., 2014, 2017). An expression construct containing cDNA of *Arabidopsis* UAGPase2 (*At2g35020*) was order-made by GenScript, San Francisco, CA, United States. The construct contained a nucleotide sequence that was optimized for bacterial expression. The full coding sequence was cloned into prokaryotic expression vector pET22b+ (Novagen) in fusion with a poly-His affinity tag (at C terminus). The construct was sequenced on both strands using a primer walking strategy with unlabeled primers (Cybergene, Huddinge, Sweden) and BigDye Terminator Cycle sequencing kit (Perkin Elmer), and transformed into BL21 (DE3) *E. coli* cells. The His_6_-tagged UAGPase2 was overexpressed and purified in the Protein Expertise Platform (Chemistry Department, Umeå University), using immobilized metal (Ni^2+^) affinity chromatography. The purified UAGPase2 was subsequently snap-frozen in liquid nitrogen for storage. Details of bacteria transformation were as earlier described for plant UGPase (Meng et al., 2009a).

In Supplementary Table S1, we have summarized details of preparation, expression and purification of recombinant enzymes used in this study, along with relevant details from other studies on the same enzymes.

### Assays

The activities of UGPases, USPase and UAGPase2 were determined in the forward direction of their reactions, using an assay based on quantification of the Pi released from inorganic pyrophosphate (PPi), the product of the pyrophosphorylase reaction. Procedures followed were generally those described in Litterer et al. (2006a) and Decker et al. (2012). Assays (each in a final volume of 50 μl) were run on 96-well plates (Sarstedt, Germany) and contained 100 mM Hepes (pH 7.5), 5 mM MgCl_2_, 0.5 unit of inorganic pyrophosphatase (Roche, Switzerland), an aliquot of a purified UGPase, USPase or UAGPase2 and varied concentrations of a sugar-1-P and a NTP (detailed concentrations are given in Figure legends). Reactions were initiated by addition of a pyrophosphorylase, were run at room temperature for 12 min, and were terminated by addition of 50 μl Pi-detection solution (for final concentration: 100 mM acetate, 0.7% ascorbic acid and 1.5% ammonium molybdate). Reactions were developed at room temperature for 5 min and the absorbance at 720 nm was measured to determine the amount of the blue colored phosphate-molybdenum complex that was proportional to the amount of phosphate present. The amount of produced Pi was quantified with a Pi standard curve.

Effects of different inhibitors on UGPase, USPase and UAGPase activities were assayed in the direction of Glc-1-P and UTP formation, using a coupling enzyme system, as described in Decker et al. (2012). The assays (300 μl each) contained 100 mM Hepes (pH 7.5), 5 mM MgCl_2_, 0.3 mM NADP+, 0.5 unit Glc-6-P dehydrogenase (Roche), 0.5 unit phosphoglucomutase (Sigma Aldrich), and concentrations of UDP-Glc and PPi at their *K*_m_ values for a given enzyme: for UGPase – 0.034 and 0.039 mM, respectively (Meng et al., 2009a); for USPase – 0.3 and 0.16 mM, respectively (Litterer et al., 2006b); and for UAGPase2 0.21 and 0.32 mM, respectively, (see Supplementary Figure S5). Assays were started by addition of a pyrophosphorylase, and were carried out by monitoring Glc-1-P production via its coupling to the formation of NADPH at 340 nm using Beckman DU 530 spectrophotometer.

In all activity determinations, assays were done at least twice for each experimental point, and the variation was usually less than 10%. A unit of enzymatic activity is defined as amount of the enzyme required either to produce 1 μmol of PPi per min (forward reaction) or to produce 1 μmol of Glc-1-P per min (reverse reaction).

### QSAR Analyses

In order to extend the range of possible sugar-1-phosphates as substrates of the pyrophosphorylases, we used the QSAR approach. The QSAR was based on Free-Wilson model (Free and Wilson, 1964), which analyzes properties of compounds that were experimentally verified to be active in a given system, and provides a list of related compounds, which are likely to be active. Since the pyrophosphorylase reactions are freely reversible (Kleczkowski and Decker, 2015), for QSAR modeling we used chemical structures of nucleotide-sugars rather than corresponding active sugar-1-phosphates. These nucleotide-sugars were classified using so called 1D descriptors, such as the configuration of hydroxyl groups on C1, C3, C4 of a sugar moiety, nucleotide-type and substituents/configurations on C2 and C5 of a sugar. The contribution to the biological activity of each descriptor was estimated using Excel Solvers non-linear GRG (Microsoft, Redmond, WA, United States).

### Homology Modeling and Analyses of Substrate Binding

For analyses of plant UGPase substrate binding interactions, we used crystal structure of *Arabidopsis* UGPase1 with bound UDP-Glc (PDB code 2ICY) (McCoy et al., 2007). Homology models of *Arabidopsis* USPase and *Arabidopsis* UAGPase2 were constructed using crystal structures of *Leishmania major* USPase (with UDP-Glc bound) (PDB code 3OH4) (Dickmanns et al., 2011) and human UAGPase2 (with UDP-GlcNAc bound) (PDB code 1JV1) (Peneff et al., 2001), respectively. Modeled structures were obtained using SWISS-model (Biasini et al., 2014), and the final accepted models had a GMQE (a quality estimation which combines properties from the target-template alignment) of 0.67 and 0.71 for USPase and UAGPase2, respectively. The comparison of the active sites of UGPase vs. USPase and UAGPase2 was performed using LigPlot+ (Laskowski and Swindells, 2011).

### Chemicals

All sugar-1-phosphates, except D-Glc-1-P, fructose-phosphates and α-D-glucosamine-1-P [GlcN-1-P], were kindly provided by Dr. Motomitsu Kitaoka, from Laboratory of Enzyme Research, NARO, Ibaraki, Japan. Separate batches of Gal-1-P were also purchased from Sigma–Aldrich (Stockholm, Sweden) and from Carbosynth (Compton, United Kingdom). Glc-1-P, glucosamine-1-P (GlcN-1-P) as well as β-D-fructofuranosyl-1-P (β-D-Fruf-1-P), β-D-fructofuranosyl-2-P (β-D-Fruf-2-P) and other fructose-phosphates were from Sigma–Aldrich. Compound #41 (ZINC720558), an inhibitor of *Trypanosoma brucei* UAGPase2 (Urbaniak et al., 2013), was purchased from MolPort (Riga, Latvia), whereas cmp #6D (CID 6526371) was from (ChemBridge Inc., San Diego, CA, United States).

## Results

### Activities of UGPases with Different Sugar Phosphates and NTPs

To study substrate specificity of plant UGPases, we have used purified recombinant barley UGPase as well as UGPase1 and UGPase2 from *Arabidopsis*. The *Arabidopsis* enzymes were the first UGPase isozymes which had been characterized from a single plant species, and they had remarkably similar physical and kinetic properties (Meng et al., 2008). Each of the enzymes was screened for activity against 55 substrate combinations (11 sugar-1-phosphates and 5 different NTPs). The reasons for this wide choice of potential substrates were twofold: (i) to identify/verify the mechanisms of synthesis of nucleotide sugars reported for plant extracts (Bar-Peled and O’Neill, 2011), and (ii) to rationalize a structure-activity relationship for sugar-moiety of a nucleotide sugar with respect to substrate binding sites of distinct pyrophosphorylases (see below).

**FIGURE 1:**
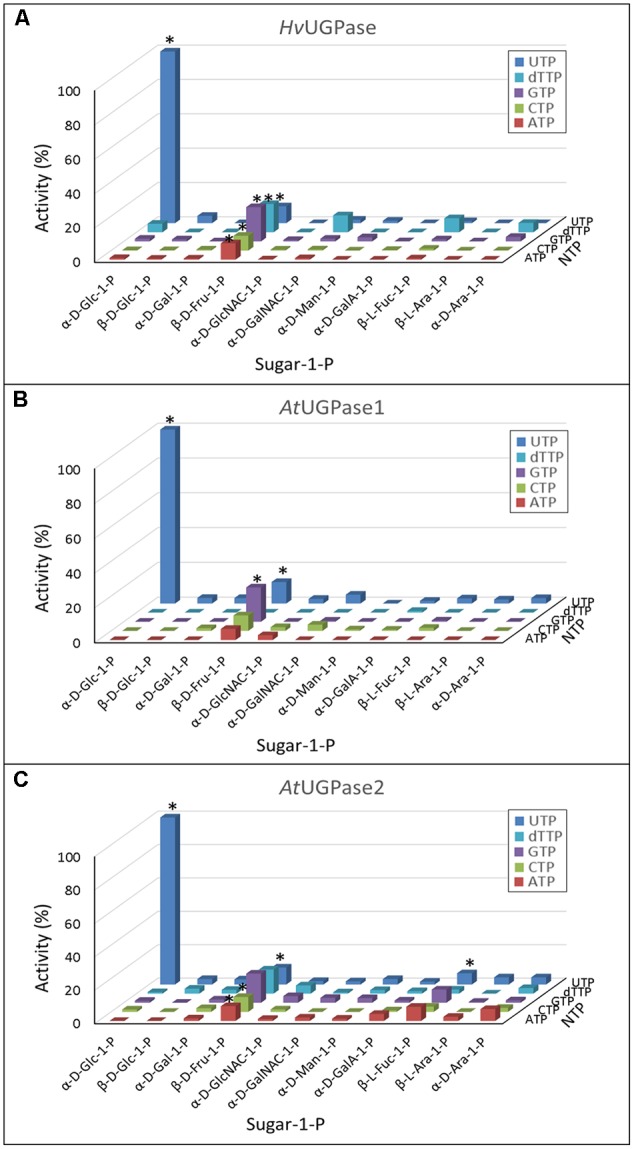
**(A–C)** Substrate specificity of barley UGPase as well as *Arabidopsis* UGPase-1 and UGPase-2. Each assay contained a sugar-1-P (at 1 mM) and a nucleoside triphosphate (at 1 mM). 100% of activity refers to 1100 units/mg protein for barley UGPase and to 29 and 11 units/mg protein for *Arabidopsis* UGPase1 and UGPase2, respectively. ^∗^ indicates significant (Student’s test) differences from background (*n* = 2, *p* < 0.05).

The assays revealed that plant UGPases are highly specific for Glc-1-P and UTP as substrates (**Figure 1**), as earlier reported (Nakano et al., 1989; Kimura et al., 1992; Ritter et al., 1996; Kleczkowski et al., 2004; Meng et al., 2008). However, the three UGPases reacted also with Fru-1-P, using a variety of NTPs as a second substrate (**Figure 1**). These activities were in the range of 7–20% of those seen with Glc-1-P and UTP. The Fru-1-P-dependent activity, although low, could be increased at least 2-fold upon increasing Fru-1-P from 1 to 5 mM (data not shown), suggesting a low affinity for this substrate. Indeed, the determined *K*_m_ values of barley UGPase and *Arabidopsis* UGPase1 with Fru-1-P were very high (Supplementary Figure S1) and difficult to determine accurately, as Fru-1-P at and above 3 mM caused clouding in the assay. This prevented assays at high substrate concentration being performed. Based on the kinetic data, we estimate that the *K*_m_ with Fru-1-P for both enzymes was well over 10 mM.

Surprisingly, none of the UGPases showed activity with Gal-1-P. This was in contrast to our earlier study (Decker et al., 2012), where the activity of barley UGPase with Gal-1-P at 0.5 mM was 12% of that with Glc-1-P. As the pyrophosphorylase activities were tested at 1 mM concentration of each of the sugar-1-phosphates (**Figure 1**), it was still possible that Gal-1-P would be reactive at higher concentrations. Indeed, as shown in Supplementary Figure S2, barley UGPase showed traces of activity when assayed with 10 mM Gal-1-P, but the rate was at most 0.14% of that with 10 mM Glc-1-P. To address this issue further, we have tested Gal-1-P batches from three sources: Sigma–Aldrich, Carbosynth and from Dr. Motomitsu Kitaoka (Ibaraki, Japan). In all cases, the activity with Gal-1-P was exceedingly low and almost linearly correlated with concentration of this substrate (up to 10 mM tested) (Supplementary Figure S2). This suggested that the *K*_m_ with Gal-1-P was very high, likely over 10 mM. This should be compared to the UGPase activity with Glc-1-P, and the *K*_m_ with this substrate of 0.45 mM (Supplementary Figure S2).

### Activities of USPase with Different Sugar Phosphates and NTPs

**FIGURE 2:**
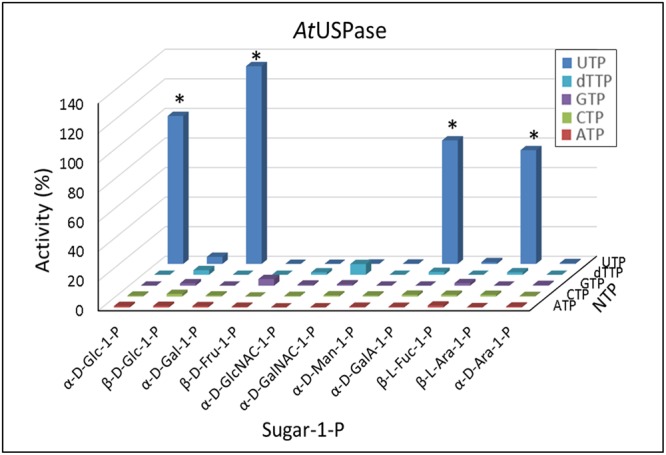
Substrate specificity of *Arabidopsis* USPase. Each assay contained a sugar-1-P (at 1 mM) and a nucleoside triphosphate (at 1 mM). 100% of activity refers to 55 units/mg protein. ^∗^ indicates significant (Student’s test) differences from background (*n* = 2, *p* < 0.05).

Compared to UGPase, the activity of USPase showed much wider substrate preferences (**Figure 2**). The enzyme was most active with Gal-1-P, but its activity with Glc-1-P, galacturonic acid-1-P (GalA-1-P) and arabinose-1-P (Ara-1-P) was at least 60% of that shown with Gal-1-P. In all cases, UTP served as a more or less specific second substrate. Whereas the *K*_m_ values with Gal-1-P, Glc-1-P, Ara-1-P, GlcA-1-P and xylose-1-P (Xyl-1-P) (the last two compounds were not tested in the present study) have already been published for *Arabidopsis* USPase (Supplementary Table S2), in the present study we determined also its *K*_m_ with GalA-1-P; this *K*_m_ was 1.3 mM (Supplementary Figure S3). Compared to already reported (also for *Arabidopsis* USPase) *K*_m_ values with GlcA-1-P of about 0.1 mM (Litterer et al., 2006b; Kotake et al., 2007) (Supplementary Table S2), this implies that USPase has considerably higher affinity for GlcA-1-P, when compared to that for GalA-1-P.

### Activities of UAGPase2 with Different Sugar Phosphates and NTPs

**FIGURE 3:**
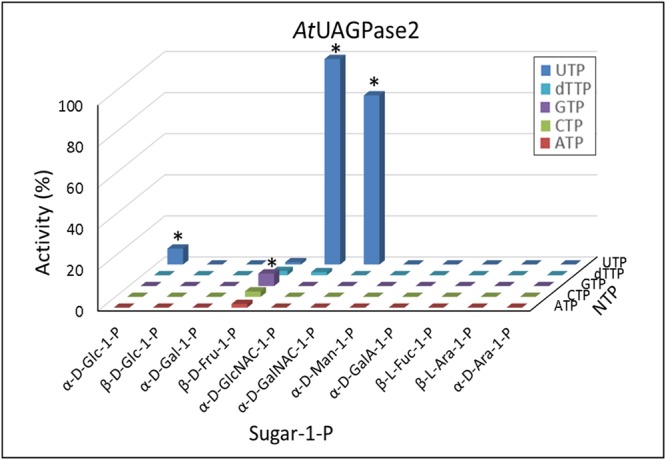
Substrate specificity of *Arabidopsis* UAGPase2. Each assay contained a sugar-1-P (at 1 mM) and a nucleoside triphosphate (at 1 mM). 100% of activity refers to 6.5 units/mg protein. ^∗^ indicates significant (Student’s test) differences from background (*n* = 2, *p* < 0.05).

Based on data in **Figure 3**, UAGPase2 reacted only with GlcNAc-1-P and GalNAc-1-P and, to some extent, with Glc-1-P. Similar to UGPase and USPase, in all cases UTP served as the only effective nucleotide donor. We have also observed very low rates with Fru-1-P, when GTP was the second substrate, but the significance of this is unclear. Earlier study on *Arabidopsis* UAGPase2 also reported highest rates with GlcNAc-1-P and GalNAc-1-P, and some activity with Glc-1-P (Yang et al., 2010), but they did not report *K*_m_ values with GalNAc-1-P nor Glc-1-P. Thus, in the present study, the determined *K*_m_ value of UAGPase2 with GalNAc-1-P was 1.0 mM (Supplementary Figure S4A), which was about 5-fold higher than that reported for *Arabidopsis* UAGPase2 with GlcNAc-1-P (*K*_m_ of 0.18 mM) (Yang et al., 2010). This suggested that GlcNAc-1-P would be the preferred *in vivo* substrate. The same conclusion can be reached when comparing *K*_m_ values with UTP when GalNAc-1-P or GlcNAc-1-P served as the second substrate: 3 mM (Supplementary Figure S4B) vs. 0.2 mM (Yang et al., 2010), respectively.

The determined *K*_m_ values for UAGPase2 with Glc-1-P and UTP (forward reaction) were 3.2 and 0.4 mM, respectively, and with UDP-Glc and PPi (reverse reaction) – 0.21 and 0.32 mM, respectively (Supplementary Figure S5). Given its relatively high *K*_m_ with Glc-1-P, *Arabidopsis* UAGPase2 is rather unlikely to contribute to UDP-Glc formation *in vivo*, especially that plants contain both UGPase and USPase which have low *K*_m_ and high activity with Glc-1-P. Since Glc-1-P is the common substrate (along with UTP) for UAGPase2, UGPase and USPase, assays of Glc-1-P/UTP-dependent activity in crude plant extracts would reflect the sum of activities of the three pyrophosphorylases. The same concerns assays of UDP-Glc/PPi-dependent activity in crude extracts.

### Predicting and Verifying other Substrates for UGPase, USPase and UAGPase2

By screening pyrophosphorylase activities against 11 sugar-1-phosphates, we found that UGPase reacted only with Glc-1-P and Fru-1-P (**Figure 1**), whereas USPase and UAGPase2 used respectively four and three of those compounds as substrates (**Figures 2, 3**). In an effort to identify other substrates, we used QSAR approach, based on Free-Wilson model (Free and Wilson, 1964). The model takes into account properties of compounds related to those that were found active in a given system (see Material and Methods) and predicts whether they would be active as well solely based on similarity to active compounds. In our analysis, different configurations and types of substitutions to a nucleotide-sugar molecule (mainly sugar) were scored, allowing a simplified prediction of the activity of a given derived nucleotide-sugar with a given pyrophosphorylase.

For UGPase, which was only active with Glc-1-P and, to some extent, Fru-1-P (**Figure 1**), no new potential substrates could be predicted. However, since the reactivity of UGPase with Fru-1-P was surprising, other fructose-phosphates were selected, based on commercial availability, and tested as potential substrates. Thus, in a separate study, the activity of barley UGPase was assayed with 1 mM concentrations of Fru-1-P, Fru-2-P, Fru-6-P, and Fru-2,6-bisP. The results demonstrated that, in addition to Fru-1-P, the UGPase had considerable activity (27% compared to the rate with Glc-1-P) with Fru-2-P (Supplementary Figure S6). Similar to Fru-1-P, the activity with Fru-2-P was characterized by a very high *K*_m_ (over 10 mM) (Supplementary Figure S6B).

**FIGURE 4:**
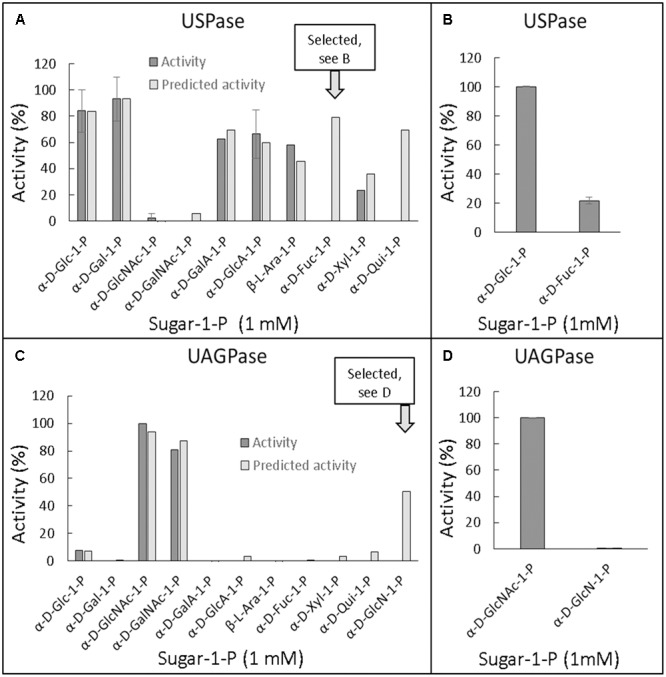
Quantitative structure activity relationship (QSAR) of *Arabidopsis* USPase **(A,B)** and *Arabidopsis* UAGPase2 **(C,D)** with different sugar-1-phosphates as substrates. **(A,C):** Determined (also literature-based, referenced in Supplementary Table S2) data for USPase and UAGPase activity, respectively, were used to construct a model (Free and Wilson, 1964) for possible other substrates, using 1D-descriptors (see Materials and Methods). **(B,D)**: Verification of the predicted substrate activity. All assays, done in two replicates, contained 1 mM sugar-1-P and 1 mM UTP.

For USPase, the QSAR analysis predicted that α-D-fucose-1-P (α-D-Fuc-1-P) and α-D-quinovose-1-P (Qui-1-P) could serve as substrates for this enzyme (**Figure 4A**). Since Qui-1-P is not commercially available, in subsequent studies we could test only the other compound. Assays of USPase with α-D-Fuc-1-P revealed that this compound indeed served as substrate, and the activity was about 20% of that with Glc-1-P (**Figure 4B**). The reactivity of USPase with α-D-Fuc-1-P was earlier inferred by Liu et al. (2013) who used *Arabidopsis* USPase as one of three “coupling enzymes” to produce UDP-α-D-Fuc from α-D-Fuc. In the present study, by directly measuring USPase activity with α-D-Fuc-1-P, we were able to determine *K*_m_ for this compound (Supplementary Figure S3). The *K*_m_ was relatively high at 3.4 mM.

The QSAR analyses of possible alternative substrate(s) for UAGPase2 revealed only GlcN-1-P as a plausible candidate (**Figure 4C**). However, assays of UAGPase2 with this compound yielded no activity (**Figure 4D**). This probably underscores the importance of the acetyl group and/or the secondary amine in GlcNAc-1-P, one of true substrates of plant UAGPase. The lack of activity with GlcN-1-P was also reported for UAGPase from *Aspergillus fumigatus* (a fungus) (Fang et al., 2013).

### Structure-Function Relationships for Sugar-1-Phosphates as Substrates of UGPase, USPase and UAGPase2

**FIGURE 5:**
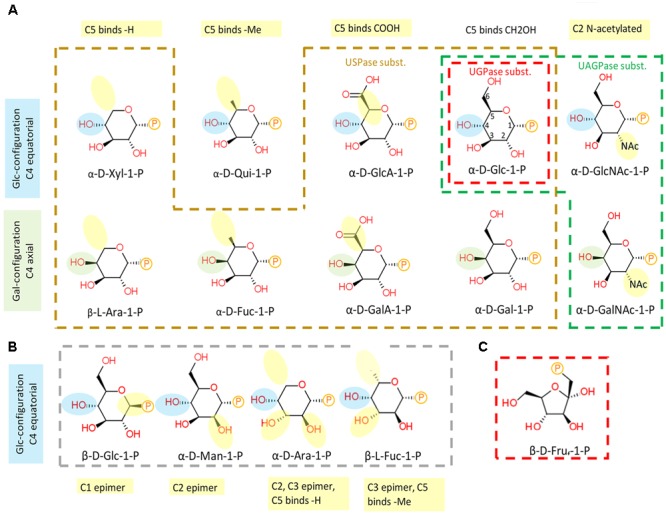
Substrate structure-activity relationship of plant UDP-sugar producing pyrophosphorylases. **(A)** Sugar-1-phosphates which are substrates for respective enzymes are encircled in different colors: UGPase - red, USPase – orange, and UAGPase2 – green. **(B)** Inactive compounds are encircled in gray. **(C)** Fru-1-P drawn in furanose form. Numbering of C-atoms are shown on α-D-Glc-1-P. Phosphate group is shown as P. Blue and green highlights of –OH at C4 refer to equatorial (Glc-like) and axial (Gal-like) configurations, respectively. Yellow highlight refers to modifications in relation to Glc.

The chemical structures of sugar-1-phosphates considered in this analysis are shown in **Figure 5**. The sugars were divided into those in the pyranose and furanose (only Fru-1-P) form, and listed according to orientation of the hydroxyl group at carbon 4 (C4) (equatorial or axial) and according to whether they were active or inactive with any of the UDP-sugar producing pyrophosphorylases. Please note that Fru-1-P is presented here as β-D furanose (**Figure 5C**), but the exact structure of this compound in its active form in solution, i.e., whether it is in α or β configuration and/or whether in furanose or pyranose form, is unknown. The list includes also Qui-1-P, which was suggested by the QSAR model to serve as one of USPase substrates (**Figure 4A**), but was not tested. Omitted from the diagram were certain C3 epimers of Glc-1-P (allose-1-P, altrose-1-P, gulose-1-P, idose-1-P and talose-1-P) which are not commercially available and for which there are no described roles in plants.

As shown in **Figure 5A**, UGPase accepts phosphorylated hexoses which have a Glc-type configuration of hydroxyl groups at C1-4 and C6, but allowing also for a trace activity with Gal-1-P, a C4 epimer of Glc-1-P. Concerning USPase, which can react with several phosphorylated hexoses and pentoses as substrates, its specificity is likely determined by the equatorial hydroxyl group at C2 of the sugar molecule (**Figure 5A**). This requirement could be further verified if the reactivity with Qui-1-P were tested. In comparison to UGPase and USPase, UAGPase2 reacts only with phosphorylated hexoses with an *N*-acetyl group at C2 of Glc or Gal moieties, but can also react with Glc-1-P (but not Gal-1-P), where the *N*-acetyl moiety at C2 is replaced by a hydroxyl group (**Figure 5A**). The sugars that were inactive for all three pyrophosphorylases are epimers of the active sugars, and they differ from them in the stereochemical attachment of hydroxyl groups at either C1, C2, or C3 position (**Figure 5B**).

### Conservation of UDP-Sugar Binding Sites in Plant UGPase, USPase and UAGPase2

**FIGURE 6:**
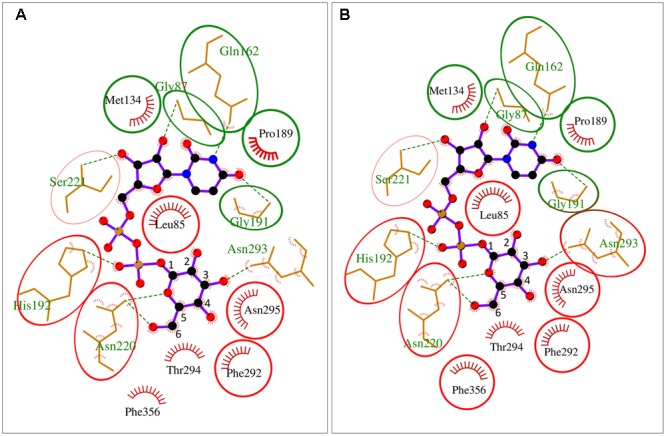
Schematic representation of UDP-Glc binding to *At*UGPase1 vs. *At*USPase **(A)** and *At*UGPase1 vs. *At*UAGPase2 **(B)**. Names of aa which interact or are in proximity of UDP-Glc are in black (hydrophobic interaction) or green font (hydrogen bond). Numbers for aa refer to UGPase aa sequence. Green circles mark the same type of aa residues in the comparable position for all three enzymes. Red circles mark aa which have an equivalent aa counterpart for a given UGPase/USPase and UGPase/UAGPase comparison. The uncircled aa are only found in *At*UGPase. Active sites of the pyrophosphorylases were analyzed and presented using LigPlot+, based on the resolved crystal structure of *At*UGPase1 (2ICY) and homology models of *At*USPase and *At*UAGPase2, based on crystal structures of *Leishmania* USPase (PDB 3OH4) (Dickmanns et al., 2011) and human UAGPase (PDB 1JV1), (Peneff et al., 2001), respectively.

Besides their substrate specificity, we have also analyzed details of UDP-sugar binding to crystal structure of *Arabidopsis* UGPase1 (McCoy et al., 2007) and to homology-modeled structures of *Arabidopsis* USPase and *Arabidopsis* UAGPase2 bound to a high affinity substrate (UDP-Glc for UGPase and USPase; and UDP-GlcNAc for UAGPase) (Supplementary Figure S7). The homology models were respectively based on crystal structures of *Leishmania* USPase (Dickmanns et al., 2011) and human UAGPase2 (Peneff et al., 2001). As seen in Supplementary Figure S7, the relative positioning of UDP-sugar and at least some of the interacting amino acid (aa) residues (via hydrogen bonds and hydrophobically) appear similar for all three enzymes, but a more detailed comparison was needed. To do so, we have overlayed substrate binding pockets of *At*UGPase1 and *At*USPase (**Figure 6A**) and those of *At*UGPase1 and *At*UAGPase2 (**Figure 6B**). To simplify representations of the active sites, only *At*UGPase1 with bound UDP-Glc is shown, but aa residues in an equivalent position in the compared structure (USPase or UAGPase) are encircled in red. Also, the numbers for aa reflect those of UGPase aa sequence.

In both UGPase/USPase and UGPase/UAGPase cases, there appears to be high degree of conservation of aa residues interacting with the nucleotide part of UDP-sugar. All three pyrophosphorylases contain amino acid residues corresponding to Gly87, Met134, Gln162 Pro189 and Gly191 of UGPase in equivalent positions, which may interact/ stabilize the uridine portion of the UDP-sugar substrate. In some other cases shown in **Figure 6**, the three pyrophosphorylases share similar aa (but not identical) in equivalent positions which may be involved in stabilizing/interacting with given parts of the UDP-sugar substrate. For instance, ribose part of uridine is stabilized/ interacts with either a leucine residue (Leu85 and Leu130 for UGPase and UAGPase2, respectively) or with valine (Val134 for USPase) (Supplementary Figure S7).

Residues in proximity of the sugar moiety are less conserved when comparing UGPase, USPase and UAGPase2. They include, for instance, an Asn-X-Asn motif in the proximity of the C2 and/or C3 of the sugar substrate of UGPase and USPase, but not UAGPase2 (**Figure 6**). Also, His192 and His 254 in UGPase and USPase, respectively, occupy the space in which Asn250 is present in UAGPase2, i.e., in the proximity of the diphosphate and -NAc portions of the nucleotide sugar substrate (Supplementary Figure S7). For UGPase, the hydroxyl group at C4 of the substrate lies in proximity of a bulky aromatic non-polar residue (Phe292), while in USPase or UAGPase2 it is located near a smaller aliphatic non-polar residue (Ile319 or Val316, respectively). Both Gly320 and Gly317 in USPase and UAGPase2, respectively, may form a hydrogen bond to the C4 hydroxyl of the sugar moiety and have no equivalent in UGPase (Supplementary Figure S7).

It should be emphasized that details of UDP-sugar binding to plant USPase and UAGPase2 in **Figure 6** are only approximate since their structures were modeled respectively on *Leishmania* and human proteins, which are evolutionarily distant from the corresponding plant proteins. For instance, *Leishmania* and *Arabidopsis* USPases share only 37% identity, based on their aa sequences (Kleczkowski et al., 2011a), whereas human and *Arabidopsis* UAGPase proteins have about 43% identity (Yang et al., 2010). The plant UGPase model, however, is based on crystal structure of *Arabidopsis* UGPase1, and thus represents a more accurate representation of the UDP-Glc binding to the enzyme. It should also be noted that these comparisons are based on rigid structures and do not take into account any local conformational changes that may occur during substrate binding and/or catalysis.

### Inhibitor Effects on Purified Enzymes

In recent study (Decker et al., 2017), we have separately used purified UGPase and purified USPase to screen a chemical library for compounds affecting a given activity. Surprisingly, the identified componds inhibited both UGPase and USPase activities, probably reflecting similar aspects of active sites of those proteins (Kleczkowski et al., 2011b). Hit expansion analyses for one of the compounds, a salicylate derivative, yielded an analog, named cmp #6D, which acted as efficient inhibitor of both enzymes, and was also active *in vivo* by inhibiting pollen germination (Decker et al., 2017). Since homology-modeled *At*UAGPase2 tertiary structure is in many aspects similar to crystal structures of UGPase and USPase (McCoy et al., 2007; Yang et al., 2010; Dickmanns et al., 2011; Kleczkowski et al., 2011b), we tested whether cmp #6D had any effect on activity of purified *Arabidopsis* UAGPase2. Indeed, cmp #6D at 50 μM inhibited UAGPase2 activity by 50% (**Table 1**), and the degree of inhibition was roughly comparable with that for UGPase and USPase.

**Table 1:** Inhibition of purified barley UGPase, *Arabidopsis* USPase and *Arabidopsis* UAGPase2 by cmp #6D and #41.

	Activity (%)		
Enzyme	No inhibitor	cmp #6D	cmp #41
UGPase	100 ± 2	37 ± 2	91 ± 8
USPase	100 ± 2	56 ± 6	96 ± 6
UAGPase2	100 ± 2	50 ± 8	100 ± 12

*Assays were carried out in the pyrophosphorylytic direction (see Material and Methods). The 100% activities for UGPase, USPase and UAGPase corresponded to 405, 12 and 7 units/mg, respectively. Inhibitors were at 50 μM. Data are mean ± SE (*n* ≥ 2).*

In addition to effects of cmp #6D, we have also tested UAGPase2 sensitivity to an indolinone-derivative which was earlier identified as inhibiting UAGPase from *Trypanosoma brucei* (with Ki of 60 μM), but not human UAGPase (Urbaniak et al., 2013). This inhibitor (which we called cmp #41) had no effect on *Arabidopsis* UAGPase2 activity, even at a concentration as high as 0.4 mM (data not shown), nor on activities of barley UGPase and *Arabidopsis* USPase (**Table 1**). Until the crystal structure of *Arabidopsis* UAGPase2 is resolved, it is unknown whether the lack of inhibition by cmp #41 is the result of any particular structural difference between the *Trypanosoma* and *Arabidopsis* UAGPase2 proteins.

## Discussion

In photosynthetic tissues, pyrophosphorylases represent the most important mechanism of the production of a UDP-sugar, as they use UTP and sugar-1-P that are derived more or less directly from photosynthetic light reactions and the Calvin cycle, respectively. In sink tissues, the pyrophosphorylases predominantly use sugar-1-P derived from hydrolysis of sucrose by either SuSy or invertase. In those tissues, SuSy may have a more prominent role, since it uses transported/accumulated Suc to produce UDP-Glc as one of its substrates (Huber and Akazawa, 1986; Kleczkowski, 1994b), which then serves as direct precursor to e.g., cellulose formation (Fujii et al., 2010). In cereal seed endosperm, the SuSy-derived UDP-Glc may also provide a metabolic link between sucrose hydrolysis and starch synthesis, being used in the reverse reaction of UGPase to produce Glc-1-P, which then serves as substrate for cytosolic AGPase (Kleczkowski, 1996). Whereas many UDP-sugars can be derived from UDP-Glc via nucleotide sugar interconversion reactions (e.g., UDP-Glc epimerase or UDP-Glc dehydrogenase) (Kleczkowski and Decker, 2015), the exact contributions of these different reactions to the nucleotide-sugar pools are still unclear, although it appears that *de novo* pathways dominate (Sharples and Fry, 2007). The role of USPase has already been demonstrated in *Arabidopsis* where it participates in the metabolism of UDP-Ara and UDP-Xyl, based on analyses of transgenic plants with knocked-down USPase activity (Geserick and Tenhaken, 2013a,b). In addition, these and other studies on transgenic plants with impaired/knocked out USPase, UGPase and UAGPase activities (e.g., Schnurr et al., 2006; Kotake et al., 2007; Meng et al., 2009b; Park et al., 2010; Chen et al., 2014) have underlined the importance of these enzymes during reproductive phases of plant development, in most cases by affecting cell wall polysaccharide formation (reviewed in Kleczkowski and Decker, 2015).

### Revisiting Substrate Specificity of UDP-Sugar Producing Pyrophosphorylases

Barley and *Arabidopsis* UGPases as well as *Arabidopsis* USPase and *Arabidopsis* UAGPases have been the most extensively studied representatives of the plant UDP-sugar producing family (Ritter et al., 1996; Kleczkowski et al., 2004, 2011a; Litterer et al., 2006b; Meng et al., 2008; Yang et al., 2010) and thus we used them as representative enzymes to reexamine substrate specificity of this group of enzymes. Moreover, this is the first study where activities and kinetics of purified UGPases, USPases and UAGPase are examined side by side, using similar assay systems.

Both barley UGPase and the two *Arabidopsis* UGPases had very similar substrate specificity, with Glc-1-P and UTP acting as the most active substrates, but they had also a 7–20% activity with Fru-1-P, regardless of the NTP used (**Figure 1**). This reactivity with different NTPs, not only UTP, was unique for Fru-1-P and the UGPases, and was not observed for any other sugar-1-P serving as substrate for either UGPase, USPase or UAGPase. The Fru-1-P- and UTP-dependent activity was earlier reported for an isozyme/isoform of potato tuber UGPase (from a cold-sweetening resistant cultivar) (Gupta and Sowokinos, 2003).

The Fru-1-P-dependent activity was observed for the UGPases, but not for USPase nor UAGPase activities (**Figures 2, 3**). Interestingly, a USPase from a bacterium *Thermus caldophilus* was also shown not to have any activity with Fru-1-P, but it was activated by this compound (Kim et al., 1999). The *K*_m_ of barley UGPase with Fru-1-P was high (over 10 mM) (Supplementary Figure S1), so it is likely that Glc-1-P (*K*_m_ of 0.33 mM; Decker et al., 2012) would largely outcompete Fru-1-P as an *in vivo* substrate. The origin of Fru-1-P in plants is also problematic, even though this compound was found in several plant species (e.g., Graham and ap Rees, 1965; Geigenberger et al., 2004), similar to UDP-Fru (Feingold, 1982). Fru-1-P can be produced either from Fru by a ketohexokinase, a distinct type of hexokinase active in animal liver (Geigenberger et al., 2004), or by an aldolase (B-type), also found in liver, which uses glyceraldehyde and dihydroxyacetone phosphate as substrates, or by a phosphofructomutase-like activity, producing Fru-1-P from Fru-6-P, as reported for *Aeromonas hydrophila*, a species belonging to Proteobacteria (Binet et al., 1998). To our knowledge, neither of these enzymes are present in plants, suggesting yet unknown mechanism of Fru-1-P formation there.

UGPase was also active with Fru-2-P, again with a high *K*_m_ (over 10 mM) (Supplementary Figure S6). Similar to UDP-(1)-Fru, the rationale for producing and usage of UDP-(2)-Fru *in vivo* is obscure. On the other hand, chemical analyses of UDP-Fru extracted from Jerusalem artichoke suggested that most of it is in the UDP-(2)-Fru form rather than that of UDP-(1)-Fru (Taniguchi and Nakamura, 1972). Fru-(2)-glycosidic bonds are found in some naturally occurring molecules, e.g., sucrose (Daudé et al., 2012). UDP-2-Fru would resemble the UDP-sugars used by glycosyltransferase reactions (Lairson et al., 2008), as the reported nucleotide sugars, both furanoses and pyranoses, are commonly activated on anomeric carbon (e.g., by pyrophosphorylases) (Bar-Peled and O’Neill, 2011). Fru-2-P, the substrate for formation of UDP-(2)-Fru by UGPase, could originate from C-6 dephosphorylation of Fru-2,6-bisP; such an activity has been reported in both plant and yeast extracts (Larondelle et al., 1989).

The reactivity of UGPase with fructose-phosphates requires further studies. Structures of products of this reaction need to be determined, using, e.g., mass and NMR spectrometry, to address the question which of the sugar carbons is activated during the reaction. An artefactual phosphatase activity of UGPase, but not USPase nor UAGPase2, in the presence of certain fructose-phosphates is also a possibility.

Unlike in our previous report (Decker et al., 2012), the activity of barley UGPase was negligible with Gal-1-P (Supplementary Figure S2). This difference could be ascribed to the fact that in the earlier study we had used different batch of Gal-1-P, and we cannot rule out that it was contaminated by Glc-1-P. Based on our present results, using batches of Gal-1-P from three different sources, the results strongly indicate that the Gal-1-P-dependent activity of UGPase is unlikely to have any significance *in vivo*, due to negligible rates and very high *K*_m_ for this compound. Extremely low activities with Gal-1-P were also reported for *Arabidopsis* UGPase1 and UGPase2 isozymes (Meng et al., 2008). In contrast to UGPase, USPase can effectively carry out the same reaction at a much lower concentration of Gal-1-P (**Figure 2**), having *K*_m_ of 0.27 mM (Kotake et al., 2007) (Supplementary Table S2). Nevertheless, activities with Gal-1-P have sporadically been reported for plant UGPases (Gupta and Sowokinos, 2003; Kim and Ahn, 2013), including the chloroplastic UGPase isozyme (Okazaki et al., 2009).

Previous studies of non-recombinant barley UGPase (purified from barley malt) (Ritter et al., 1996) demonstrated that the enzyme had a somewhat less strict substrate specificity (i.e., low activity with GlcA-1-P and GalA-1-P, in addition to that with Glc-1-P), when compared to the recombinant (expressed from procaryote hosts) enzymes used in the present study. These differences in substrate specificity could perhaps be explained by post-translational modifications which are specific for eucaryotes/plants (Sauerzapfe et al., 2008) and/or simply by differences in assay conditions, e.g., 1 vs. 2 mM NTP and 5 vs. 10 mM MgCl_2_ for this study and Ritter’s et al., (1996), respectively. However, it should be emphasized that, in terms of substrate specificity, plant UGPases are very different from mammalian UGPases which, in addition to Glc-1-P, have relatively high activities with several other sugar-1-phosphates, producing corresponding UDP-sugars (Knop and Hansen, 1970; Ritter et al., 1996). In this respect mammalian UGPases resemble USPases. Mammals apparently lack USPase (Kleczkowski et al., 2011a), and thus their UGPases may have evolved to compensate for that by extending their range of sugar-1-phosphates used as substrates.

As in earlier studies on substrate specificity of plant USPases (Kotake et al., 2004, 2007; Dai et al., 2006; Litterer et al., 2006a,b), *Arabidopsis* USPase was found as highly promiscuous with respect to various sugar-1-phosphates as substrates, with the highest activity shown with Gal-1-P, Glc-1-P, GalA-1-P, and L-Ara-1-P (**Figure 2**). Somewhat similar properties were reported for USPases from protozoan pathogens *Leishmania* and *Trypanosoma* (Damerow et al., 2010; Yang and Bar-Peled, 2010). However, the activity with GalA-1-P for *Arabidopsis* USPase was only seldom reported (Yang et al., 2009), and its *K*_m_ value was unknown. We have now determined *K*_m_ of *Arabidopsis* USPase with GalA-1-P, as shown in Supplementary Figure S3A. This relatively low *K*_m_ of 1.3 mM is comparable to *K*_m_ of 2.3 mM determined with GalA-1-P for pea USPase (Ohashi et al., 2006), suggesting that plant USPase (in addition to UDP-GlcA epimerase) could be involved in the production of UDP-GalA *in vivo*, e.g., during recycling of GalA released during cell wall restructuring.

*Arabidopsis* USPase was reactive with α-D-Fuc-1-P (Supplementary Figure S3B), but not with β-L-Fuc-1-P (**Figure 2**). The reactivity with α-D-Fuc-1-P was inferred from QSAR analyses (**Figure 4A**) and then confirmed experimentally (**Figure 4C**). Fuc moiety can be found in cell wall polysaccharides and in sugar components of glycoproteins, and it is usually present there in the α-L-Fuc form (Scheible and Pauly, 2004; Harholt et al., 2010; Scheller and Ulvskov, 2010). The activated form of Fuc used by glycosyltransferases is believed to be GDP-β-L-Fuc, rather than UDP-α-D-Fuc, and in plants it is synthesized from GDP-α-D-mannose (GDP-α-D-Man) via combined activities of specific dehydratase and epimerase/reductase or by a bifunctional kinase/GDP-Fuc pyrophosphorylase protein that converts β-L-Fuc to β-L-Fuc-1-P, and then to GDP-β-L-Fuc (Kotake et al., 2008). The formed GDP-β-L-Fuc may then subsequently be used by an inverting-type of a glycosyltrasferase, which inverts anomeric configuration of the sugar residue upon transferring it to a donor molecule (Lairson et al., 2008).

In contrast to GDP-β-L-Fuc, very little is known about the origins and the roles of UDP-α-D-Fuc in plants. As strong USPase knock-down had no changes in cell wall nor leaf soluble fucose content (Geserick and Tenhaken, 2013a), UDP-α-D-Fuc may not be a key player in fucose cycling or cell wall formation, but perhaps may be involved in secondary metabolism. Addition of a synthetically prepared UDP-α-D-Fuc to leaf extracts was reported to lead to incorporation of the Fuc molecule to cardenolide aglycones, a group of secondary metabolites (Faust et al., 1994), but the resulting compounds were β-D-fucosylated rather than α-D-fucosylated, suggesting the involvement of an inverting-type of a glycosyltrasferase. Plants contain many such glycosyltransferases (Lairson et al., 2008), but to our knowledge none has yet been identified which carries the inversion when using UDP-α-D-Fuc as a substrate. Considering the structural similarities between α-D-Fuc and α-D-Gal (**Figure 5**), such an enzyme can perhaps be found among the UDP-α-D-Gal-utilizing glycosyltransferases. Given its relatively low *K*_m_ of 3.4 mM with α-D-Fuc-1-P (Supplementary Figure S3B), USPase may perhaps represent yet another mechanism in the pathway from Fuc to a fucosylated product molecule.

*Arabidopsis* UAGPase2 was found to react preferentially with GlcNAc-1-P and GalNAc-1-P, with UTP acting as the sole NTP substrate (**Figure 3**), confirming earlier data on this enzyme (Yang et al., 2010). In this respect, *Arabidopsis* UAGPase2 is similar to human UAGPase (Peneff et al., 2001), but not to UAGPases from *Aspergillus fumigatus* (a fungus) and *Trypanosoma brucei* (single-celled eukaryotic pathogen), which do not react with GalNAc-1-P as substrate (Stokes et al., 2008; Fang et al., 2013). Because of its relatively low *K*_m_ values with GlcNAc-1-P (0.18 mM, Yang et al., 2010) and GalNAc-1-P (1.0 mM, Supplementary Table S2), it appears that UAGPase2 is the key activity “activating” both of these *N*-acetyl hexose-amines, so they can be used for glycosylation events in plants (Bar-Peled and O’Neill, 2011). However, the enzyme had a relatively high *K*_m_ for UTP (3 mM) when GalNAc-1-P served as the second substrate (Supplementary Figure S4), which should be compared with *K*_m_ of 0.2 mM for UTP when GlcNAc-1-P was the other substrate (Yang et al., 2010). This implies that the GlcNAc-1-P-dependent activity would be favored over that with GalNAc-1-P. This is consistent with earlier data for the reverse reaction of *Arabidopsis* UAGPase2, where *K*_m_ value with UDP-GlcNAc was over 10 times lower than that with UDP-GalNAc (0.07 mM vs. 0.81 mM, respectively) (Yang et al., 2010). To assure sufficient production of the latter, plants may have an additional mechanism in the form of an epimerase which directly converts UDP-GlcNAc to UDP-GalNAc (Zhang et al., 2006; Furo et al., 2015).

The UAGPase2 enzyme was also able to utilize Glc-1-P as a substrate (**Figure 3**). It is, however, rather unlikely to be involved in a large scale UDP-Glc synthesis *in vivo*, given its relatively low rates (Yang et al., 2010) and high *K*_m_ of 3.2 mM with this compound (Supplementary Figure S5) and, even more so, because both UGPase and USPase have much higher affinities for Glc-1-P as substrate (Supplementary Table S2). On the other hand, the relatively low *K*_m_ values for substrates of the reverse reaction (Supplementary Figure S5) make it feasible for UAGPase2 to be involved in Glc-1-P formation from UDP-Glc, similar to UGPase and USPase reaction.

*K*_m_ values determined with Glc-1-P for the three pyrophosphorylases (Supplementary Table S2) should be compared with Glc-1-P concentrations found in the cytosol of plant tissues, i.e., 0.07 mM for barley endosperm (Tiessen et al., 2012) and 0.05 mM in the cytosol of developing potato tubers (Farré et al., 2001). To our knowledge, there are no data on cytosolic concentrations of other sugar-phosphates, but they may be on the same order of magnitude as Glc-1-P, since their metabolism is frequently directly or indirectly linked to that of Glc-1-P (Kleczkowski and Decker, 2015). Thus, given *K*_m_ values for sugar-1-phosphates on the order of 0.1–10 mM (Supplementary Table S2), the *in vivo* activities of the pyrophosphorylases are probably very sensitive to even small changes in internal sugar-1-P concentration.

Concerning cytosolic UTP concentration, it was calculated at 0.23 mM for barley endosperm (cytosolic ATP at 0.47 mM) (Tiessen et al., 2012) and 0.4 and 0.7 mM for potato tubers (Farré et al., 2001) and spinach leaves (Dancer et al., 1990), respectively. Thus, the activity of UAGPase2 with GalNAc-1-P, will very much depend on even small changes of cytosolic UTP concentration (*K*_m_ of 3 mM) (Supplementary Figure S4), whereas such changes will have a lesser impact on UAGPase2 activity with Glc-1-P (*K*_m_ with UTP of 0.4 mM) (Supplementary Figure S5). For UGPase and USPase, the *K*_m_ values with UTP (when Glc-1-P was saturating) were 0.08–0.25 mM (Meng et al., 2008; Decker et al., 2012) and 0.08–0.19 mM (Litterer et al., 2006b; Kotake et al., 2007; Kleczkowski et al., 2011a), respectively, and thus those enzymes probably operate at saturating concentrations of UTP.

**FIGURE 7:**
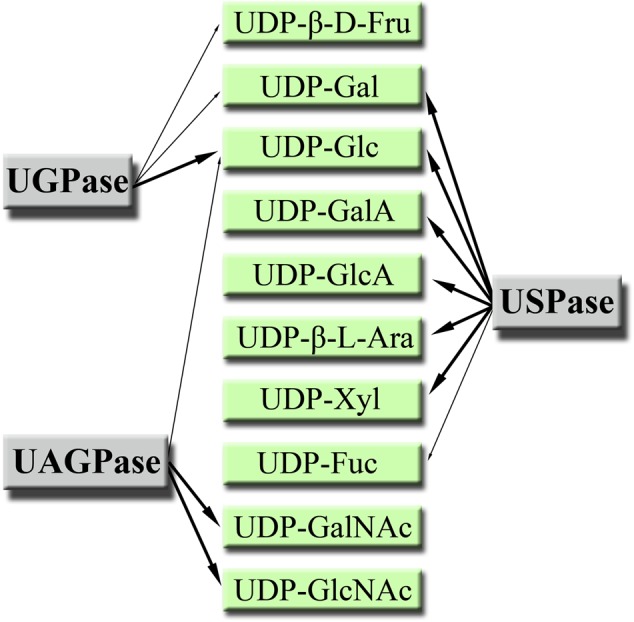
Products of plant UGPase, USPase and UAGPase2 reactions. All UDP-sugars are, unless otherwise stated, in α-D form. Major products are pointed out with thick arrows.

Products of the UGPase, USPase and UAGPase2 reactions have been summarized in **Figure 7**. Because of high costs or commercial unavailability of the respective sugar-1-phosphates, we could not study the formation of UDP-Xyl or UDP-GlcA; however, those UDP-sugars were earlier shown to be produced by *Arabidopsis* USPase (see Supplementary Table S2) and are included in **Figure 7**. Based on this summary, it appears that the three pyrophosphorylases may decisively contribute to the synthesis of a wide range of UDP-sugars which then can be taken up as substrates by a multitude of glycosyltransferase reactions in the cell.

It seems interesting to note that plant cell wall polysaccharides, which are the most abundant components of biomass in nature (Kotake et al., 2010), are composed of at least 14 different monosaccharide moieties, most of them derived from UDP-sugars (Scheible and Pauly, 2004). As cell walls contain no GlcNAc nor GalNAc as components, it appears that among the UDP-sugar producing pyrophosphorylases it is UGPase and USPase which contribute the most to the cell wall composition. On the other hand, UAGPase may have a role in providing UDP-GlcNAc as substrate for specific glycosyltrasferases responsible for posttranslational modification of proteins with *O*-GlcNAc (Olszewski et al., 2010). Also, many of the *N*-linked complex glycans terminate with GlcNAc (Rayon et al., 1999), and glycoprotein *N*-glycans may contain core GlcNAc dimers (Vanholme et al., 2014), which again would require UDP-GlcNAc as substrate for their formation. The role of UDP-GalNAc is less clear since plants lack the machinery to form mucin-type (GalNAc based) glycosylations, even though there have been reports of a mucin-type glycosylations in some algal and plant species (Niemann et al., 2015). However, when the whole machinery of mucin-type glycosylation (target-peptide, transporters and glycosyltransferase) was introduced to tobacco, the endogenous UDP-GalNAc (could originate from UDP-GlcNAc epimerase and/or UAPGase activities) was not sufficient to support GalNAc addition to the target-peptide (Daskalova et al., 2010). This suggested that the capacity for UDP-GalNAc formation in wild-type plants is low.

### UGPase, USPase and UAGPase2 Share Common UDP-Binding Active Site Architecture but Differ in Determinants of Sugar-Binding

In an attempt to rationalize the observed substrate specificities, we analyzed details of substrate binding to each of the three pyrophosphorylases. To do so, we focused on aa residues which bind/ stabilize UDP-Glc, a common product of each of the three pyrophosphorylases when reacting with Glc-1-P and UTP. The analyses yielded two structural models of UDP-Glc binding for UGPase vs. USPase (**Figure 6A**) and for UGPase vs. UAGPase2, respectively (**Figure 6B**). For binding of the nucleotide portion of UDP-sugar, the models were basically identical yielding a number of conserved aa which were common for the three pyrophosphorylases. This was not surprising, given high specificity of all three pyrophosphorylases for UTP as nucleotide donor for sugar activation reaction (**Figures 1–3**).

On the other hand, there were significant differences in binding of a sugar portion of a given UDP-sugar, especially for aa close to hydroxyl groups at C4 and C6, and to some extent C2, of the sugar (**Figure 6** and Supplementary Figure S7). Substitutions and/or configuration changes to those carbons give rise to a variety of distinct sugars which are differentially recognized by either UGPase, USPase or UAGPase2 (**Figure 5**). For both USPase and UAGPase2, the primary differences from UGPase must be the presence of aa interacting with groups attached to C4 of the sugar molecule; the two enzymes have to accept both of C4 epimers (Glc and Gal), whereas UGPase accepts only Glc. For UGPase, the C4 of the substrate lies in proximity of aromatic Phe292, whereas for USPase or UAGPase2 the C4 appears close to a smaller residue, such as Ile319 and Val316, respectively. Both USPase and UAGPase2 also appear to form a hydrogen bond from a Gly residues to the C4-hydroxy group of the substrate (Supplementary Figure S7). Thus it is tempting to suggest that, for UGPase, it is the presence of Phe292 and/or the lack of key glycine residues in proximity of C4 of the substrate (Gly320 and Gly317, for USPase and UAGPase2, respectively), that contributes to specificity for Glc-1-P. Interestingly, an analogous Gly308 in *Lm*USPase (which accepts GalA-1-P as substrate) is in the proximity of C4 of the sugar, while *Tc*USPase (homology-modeled on *Leishmania* USPase, PDB code 3OH4), which does not utilize GalA-1-P, has no similar residue (Yang and Bar-Peled, 2010). Recent studies on substrate range of barley USPase (Wahl et al., 2017) revealed that it has no activity with α-D-Fuc-1-P, and thus is distinct from *Arabidopsis* USPase (Supplementary Figure S3B). Further studies of these enzymes can possibly reveal residues involved in their distinct substrate specificity.

Regarding groups attached to C5, both UGPase and UAGPase2 appear to strictly accept -CH2OH at this position (Yang et al., 2010), whereas USPase tolerates a wide range of such groups (–H, –CH3, –CH2OH and –COOH). In this respect, UAGPase2 appears to be more related to UGPase than USPase, since both enzymes share a phenylalanine residue (Phe356 and Phe425, respectively) which is in proximity of the –CH2OH attached to C5 (**Figure 6** and Supplementary Figure S7). Similar to earlier studies on *Arabidopsis* UAGPase2 (Yang et al., 2010), the enzyme accepted only Glc-type substitutions of C6 sugar moiety, e.g., in the Yang et al. (2010) study it was not reactive with XylNAc-1-P nor GlcANAc-1-P, suggesting that it has a specific binding site for the C6 sugar group. For UAGPase2, the *N*-acetyl part of UDP-GlcNAc appears to interact with Asn250 (Supplementary Figure S7C), whereas the equivalent 3D-position in both UGPase and USPase is occupied by larger side chains of His192 and His254, respectively (Supplementary Figures S7A,B). For human UGPase, the presence of an analogous bulky histidine residue (His216) at the active site has been suggested to block GlcNAc-1-P binding (Roeben et al., 2006).

Studies on other eukaryotic UGPases and USPases, both using resolved structures (Steiner et al., 2007; Dickmanns et al., 2011) and using simulations of the reaction steps (Führing et al., 2013), have revealed that flexible portions of the enzymes are also contributing/causing the differences in substrate binding. In a study by Führing et al. (2013), the complete enzymatic cycle of *Leishmani*a UGPase was assessed taking, e.g., into account both global (at the domain level) and local changes brought about upon substrate binding to the enzyme. Understanding the role of these conformational changes for substrate specificity of plant UDP-sugar producing pyrophosphorylases may be crucial to further rationalize/ understand how UGPase, USPase and UAGPase2 interact with their substrates and catalyze their reactions.

A common architecture at or nearby substrate-binding domains appears also to be a feature for bacterial nucleotide-sugar producing pyrophosphorylases, which frequently share only less than 10% identity (based on aa sequences) with their plant counterparts (Geisler et al., 2004; Kleczkowski et al., 2004). Mild random mutagenesis of a bacterial AGPase resulted in cDNA clones coding for proteins which had their substrate specificity changed to that of UGPase and UAGPase (Sohn et al., 2006). Thus, a change of few aa could bring about a fundamental change in substrate specificity for this protein. It is unknown though whether such a “directed evolution” approach would work with plant pyrophosphorylases. On the other hand, based on comparative descriptions of the active sites of UGPase, USPase and UAGPase2 (as e.g., presented in a very simplified form in **Figure 6**), a more rational strategy could be devised to alter the substrate affinity of a given NDP-sugar pyrophosphorylase. Obviously, for this to succeed, one would greatly benefit if crystal structures were available for all three plant UDP-sugar producing pyrophosphorylases. An open question in such analyses would also be whether such modifications do not compromise binding of activators (if any), or second substrates or catalysis. Understanding factors which determine substrate specificity of a given pyrophosphorylase may also aid future attempts to classify other nucleotide-sugar binding enzymes, such as the vast and biologically important glycosyltransferase family.

### UDP-Sugar Producing Pyrophosphorylases Are Affected by the Same Inhibitors

In an earlier study (Decker et al., 2017), based on results of a chemical library screening, we have demonstrated that the activities of USPase and UGPase are inhibited by the same compounds. Subsequent hit optimization of one of the inhibitors yielded cmp #6D, which was effective both with purified enzymes and in *in vivo* experiments (Decker et al., 2017). In the present study, cmp #6D was found to inhibit also UAGPase2 activity, and the extent of inhibition was similar to that observed with UGPase and USPase (**Table 1**). The fact that the three enzymes are inhibited by the same compound suggests that it interacts with a site or component which is common for UGPase, USPase and UAGPase, and may lay at, or close to, the active sites of these proteins.

In addition, the *Arabidopsis* UAGPase2 was not affected by cmp #41 (**Table 1**), which was earlier shown to inhibit UAGPase from *Trypanosoma brucei* (Urbaniak et al., 2013). Both barley UGPase and *Arabidopsis* USPase were also not affected. Based on crystal structure analyses, the *Trypanosoma* UAGPase2 protein binds cmp #41 at a unique allosteric site, which is not present in human UAGPase (Urbaniak et al., 2013). Since the structure of *Arabidopsis* UAGPase2 has not yet been resolved, it is unknown whether this protein contains such a site. However, in analogy to human UAGPase, the lack of inhibition by cmp #41 suggests that a similar allosteric site is absent for plant UAGPase2.

The use of inhibitors to study *in vivo* functions of UGPase and USPase has been recently proposed (Decker et al., 2017) to overcome problems encountered by genetical approaches, where plants deficient or lacking a given pyrophosphorylase were frequently male-sterile or otherwise impaired in their reproductive abilities (e.g., Chen et al., 2007; Kotake et al., 2007; Meng et al., 2009b; Park et al., 2010; Geserick and Tenhaken, 2013a; Chen et al., 2014). Such an approach allows to use wild-type plants and the inhibitors are likely to target the same protein(s) in different plant species (Kleczkowski, 1994a; Blackwell and Zhao, 2003). Obviously, the fact that not only UGPase and USPase, but also UAGPase, are affected by the same compounds calls for more efforts to identify *specific* inhibitors which can discriminate between the three pyrophosphorylases. To find such a specific inhibitor, analogs of cmp #6D and other previously identified inhibitors of UGPase and USPase (Decker et al., 2017) could be examined or, alternatively, a survey of virtual chemical libraries using *in silico* screening (Shoichet, 2004) could be a possibility.

## Author Contributions

LK and DD conceived the original research plans; DD and LK designed all of the experiments; DD performed all of the experiments; LK and DD analyzed the data; LK wrote the article with contributions from DD.

## Conflict of Interest Statement

The authors declare that the research was conducted in the absence of any commercial or financial relationships that could be construed as a potential conflict of interest.

## Abbreviations

aa: amino acids
AGPase: ADP-Glc pyrophosphorylase
Ara: arabinose
Fru: fructose
Fuc: fucose
Gal: galactose
GalA: galacturonic acid
GalNAc: *N*-acetylgalactosamine
Glc: glucose
GlcA: glucuronic acid
GlcN: glucosamine
GlcNAc: *N*-acetylglucosamine
Man: mannose
NTP: nucleoside triphosphate
QSAR: quantitative structure-activity relationship
Qui: quinovose
SuSy: sucrose synthase
UAGPase: UDP-GlcNAc pyrophosphorylase
UGPase: UDP-Glc pyrophosphorylase
USPase: UDP-sugar pyrophosphorylase;
Xyl: xylose
