# Supplementary material for: Substrate Specificity and Inhibitor Sensitivity of Plant UDP-Sugar Producing Pyrophosphorylases
Source: Front Plant Sci. 2017 Sep 20;8:1610. doi: 10.3389/fpls.2017.01610 (PMC5609113; doi:10.3389/fpls.2017.01610)
Supplement: Supplementary file 1 [file Table_1.PDF]

Table S1. Details of preparation, expression and purification of recombinant enzymes used in this study, along with relevant details from other laboratories on the same enzymes. Yellow color refers to enzymes prepared by the authors and studied in the present manuscript. IMAC, immobilized metal affinity chromatography; trx, thioredoxin. A star (*) refers to a coding sequence optimized for prokaryotic expression.

Enzyme	GeneID	Expr. vector	Tag	Tag position	Expr. strain	IMAC ion	Tag removal	Ref.
Hv UGPase	Q43772	pET23d+	6xHis	C-terminal	BL21 (DE3)	Ni	-	a
At UGPase1	At3g03250	pET23d+	6xHis	C-terminal	BL21 (DE3)	Ni	-	b
At UGPase2	At5g17310	pET23d+	6xHis	C-terminal	BL21 (DE3)	Ni	-	b
At USPase	At5g52560	pETtrx	6xHis-trx	N-terminal	BL21 (DE3)	Co	TEV protease	c
At USPase	At5g52560	pET32a	6xHis-trx	N-terminal	BL21(DE3)	?	Thrombin	d
At USPase	At5g52560	pET28b	6xHis	N-terminal	BL21pLysS	Ni	Thrombin	e
At UAGPase2	At2g35020*	pET22b+	6xHis	C-terminal	BL21 (DE3)	Ni	-	-
At UAGPase2	At2g35020	pET28c	6xHis	N-terminal	BL21(de3)pLysS	Ni	-	f

[a] Martz et al. (2002)
[b] Meng et al. (2008)
[c] Decker et al. (2017)
[d] Kotake et al. (2007)
[e] Litterer et al. (2006b)
[f] Yang et al. (2010)
