# Supplementary material for: Substrate Specificity and Inhibitor Sensitivity of Plant UDP-Sugar Producing Pyrophosphorylases
Source: Front Plant Sci. 2017 Sep 20;8:1610. doi: 10.3389/fpls.2017.01610 (PMC5609113; doi:10.3389/fpls.2017.01610)
Supplement: Supplementary file 2 [file Table_2.PDF]

Table S2

Summary of the K_{cat}/K_m and K_m values with sugar-1-P (using UTP as second substrate) for barley UGPase, *Arabidopsis* USPase and *Arabidopsis* UAGPase2. All sugar-1-phosphates were at the "α-D" configuration, except β-L-Ara-1-P, β-D-Fru-1-P, and β-D-Fru-2-P. The data from the present study are in bold. NA, no activity; nd, not determined.

	UGPase		USPase		UAGPase2	
**Substrate**	**k_{cat}/K_m (s^{-1} mM^{-1})**	**K_m (mM)**	**k_{cat}/K_m (s^{-1} mM^{-1})**	**K_m (mM)**	**k_{cat}/K_m (s^{-1} mM^{-1})**	**K_m (mM)**
Glc-1-P	**2052**	0.33[a], 0.26[b], **0.65**	530[c], 160[d]	0.23[c], 0.42[d]	**0.04**	**3.2**
Gal-1-P	nd	>10[a], **>10**	490[c]	0.27[c]	**NA**	**NA**
GlcA-1-P	nd	nd	710[c], 310[d]	0.09[c], 0.13[d]	NA[e]	nd
GalA-1-P	**NA**	**NA**	**43**	**1.3**	**NA**	**NA**
Xyl-1-P	nd	nd	51[c]	1[c]	nd	nd
Ara-1-P	**NA**	**NA**	92[c]	1.5[c]	**NA**	**NA**
GlcNAc-1-P	**NA**	**NA**	**NA**, NA[c]	**NA**, NA[c]	160[e]	0.18[e]
GalNAc-1-P	**NA**	**NA**	**NA**	**NA**	**16.8**	**1.0**
Fuc-1-P	nd	nd	**1.0**	**3.4**	nd	nd
Fru-2-P	nd	**>10**	nd	nd	nd	nd
Fru-1-P	nd	**>10**	**NA**	**NA**	**NA**	**NA**

[a] Decker et al. (2012)
[b] Decker et al. (2014)
[c] Kotake et al. (2007)
[d] Litterer et al. (2006b)
[e] Yang et al. (2010)
