# Supplementary material for: Substrate Specificity and Inhibitor Sensitivity of Plant UDP-Sugar Producing Pyrophosphorylases
Source: Front Plant Sci. 2017 Sep 20;8:1610. doi: 10.3389/fpls.2017.01610 (PMC5609113; doi:10.3389/fpls.2017.01610)
Supplement: Supplementary file 3 [file Image_1.PDF]

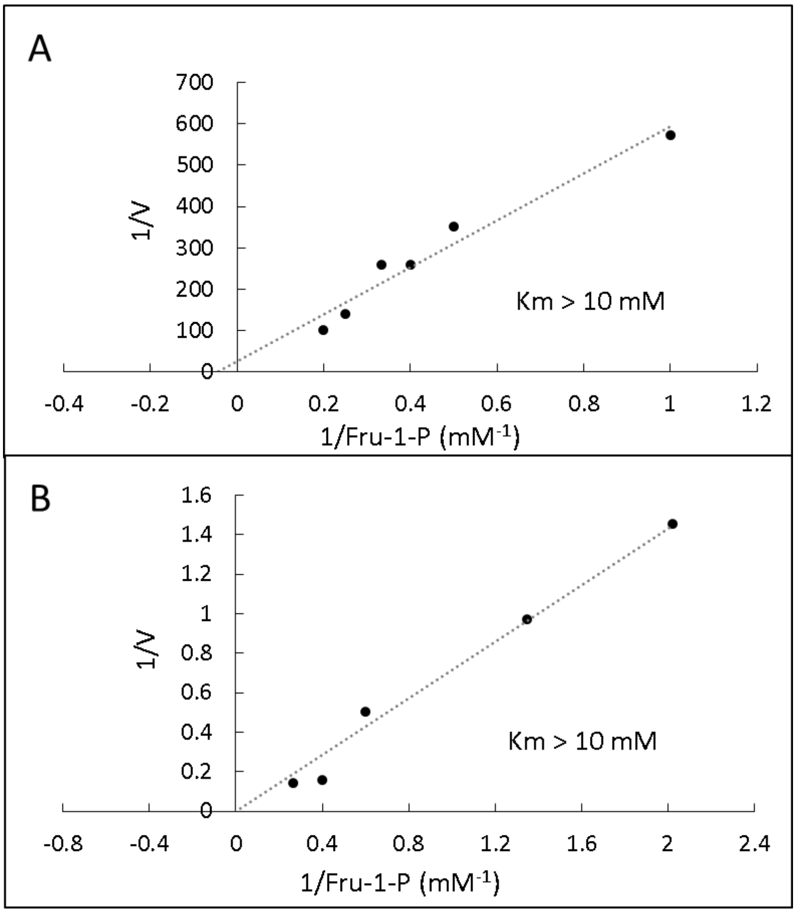

Fig. S1. Determination of K_m with Fru-1-P of *Arabidopsis* UGPase1 (A) and barley UGPase (B). UTP was kept constant at 1 mM, whereas Fru-1-P was varied from 1 to 5 mM **(A)** and from 0.5 to 3.8 mM **(B).** V, activity (units/mg protein).
