# Supplementary material for: Substrate Specificity and Inhibitor Sensitivity of Plant UDP-Sugar Producing Pyrophosphorylases
Source: Front Plant Sci. 2017 Sep 20;8:1610. doi: 10.3389/fpls.2017.01610 (PMC5609113; doi:10.3389/fpls.2017.01610)
Supplement: Supplementary file 4 [file Image_2.PDF]

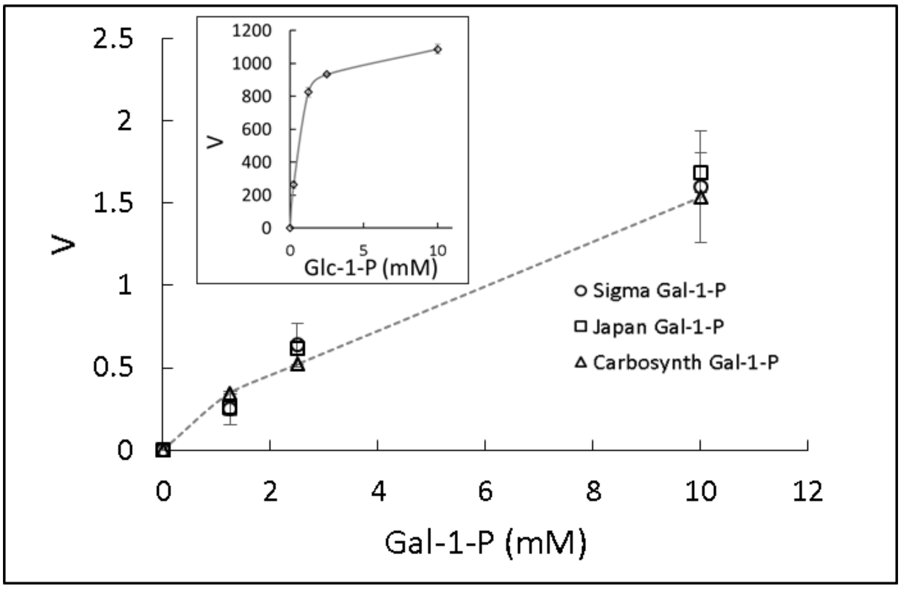

Fig. S2. Rates of barley UGPase vs. Gal-1-P from different sources. Inset represents activities with Glc-1-P, from which approximate V_{max} (1184 units/mg protein) and K_m value with Glc-1-P (0.6 mM) were calculated by fitting the data to the function V = (V_{max} [Glc-1-P])/ (K_m + [Glc-1-P]) and using the Excel Solver Add-in. Assays contained 1 mM UTP. V, activity (units/mg protein).
