# Supplementary material for: Substrate Specificity and Inhibitor Sensitivity of Plant UDP-Sugar Producing Pyrophosphorylases
Source: Front Plant Sci. 2017 Sep 20;8:1610. doi: 10.3389/fpls.2017.01610 (PMC5609113; doi:10.3389/fpls.2017.01610)
Supplement: Supplementary file 5 [file Image_3.PDF]

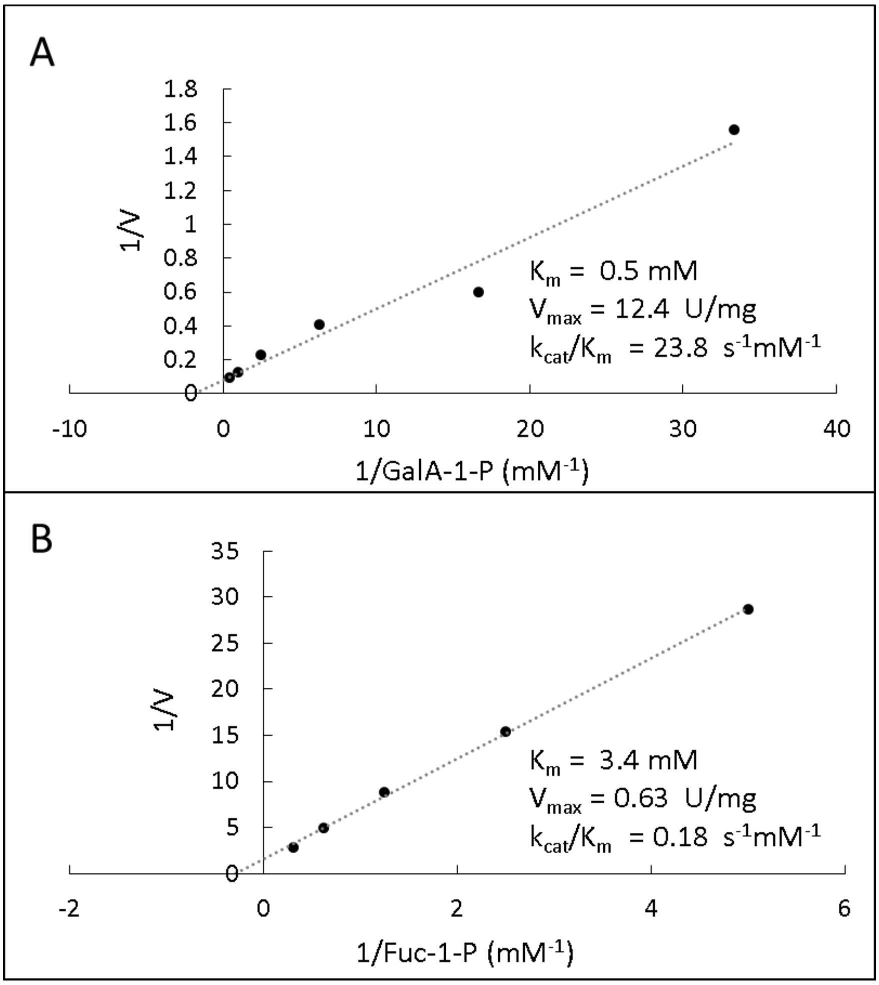

Fig. S3. K_m of *Arabidopsis* USPase with GalA-1-P (A) and with α-D-Fuc-1-P (B). In **(A)**, GalA-1-P was varied from 0.03 to 2.4 mM, whereas UTP was at 1 mM. In **(B)**, Fuc-1-P was varied from 0.2 to 3.2 mM, and UTP was at 1 mM. V, activity (units/mg protein).
