# Supplementary material for: Substrate Specificity and Inhibitor Sensitivity of Plant UDP-Sugar Producing Pyrophosphorylases
Source: Front Plant Sci. 2017 Sep 20;8:1610. doi: 10.3389/fpls.2017.01610 (PMC5609113; doi:10.3389/fpls.2017.01610)
Supplement: Supplementary file 6 [file Image_4.PDF]

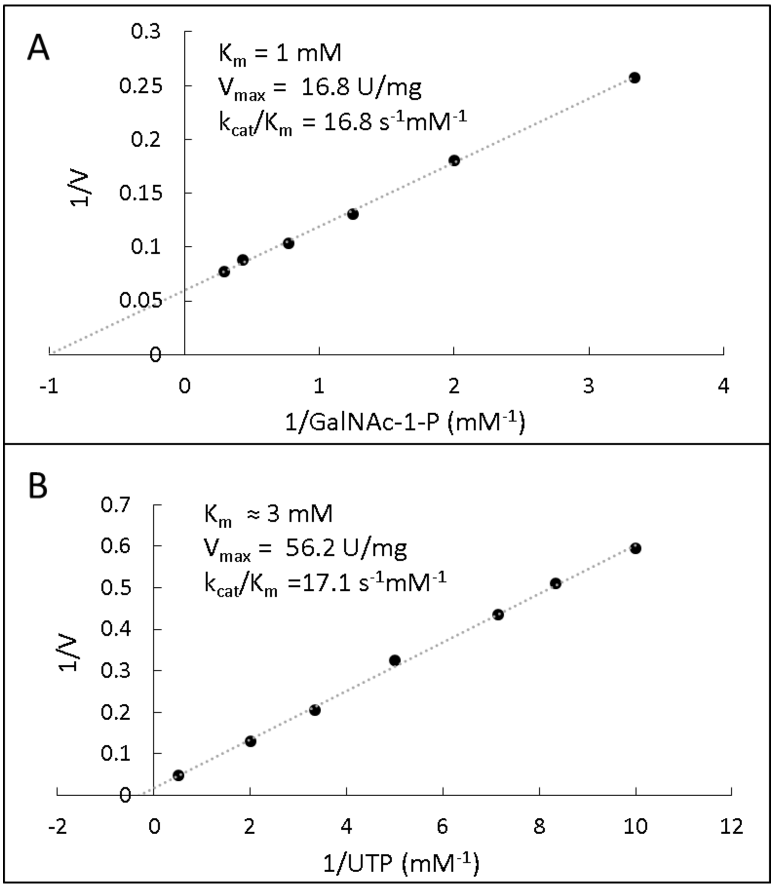

Fig. S4. K_m of *Arabidopsis* UAGPase2 with GalNAc-1-P (A) and UTP (B). In **(A)**, GalNAc-1-P was varied from 0.3 to 3.4 mM, whereas UTP was at 1 mM. In (**B**), UTP was at varied from 0.1 to 2 mM, whereas GalNAc-1-P was at 3 mM. V, activity (units/mg protein). Please note that the Km for GalNAc-1-P is an apparent Km, since it was determined at non-saturating concentration of UTP.
