# Supplementary material for: Substrate Specificity and Inhibitor Sensitivity of Plant UDP-Sugar Producing Pyrophosphorylases
Source: Front Plant Sci. 2017 Sep 20;8:1610. doi: 10.3389/fpls.2017.01610 (PMC5609113; doi:10.3389/fpls.2017.01610)
Supplement: Supplementary file 7 [file Image_5.PDF]

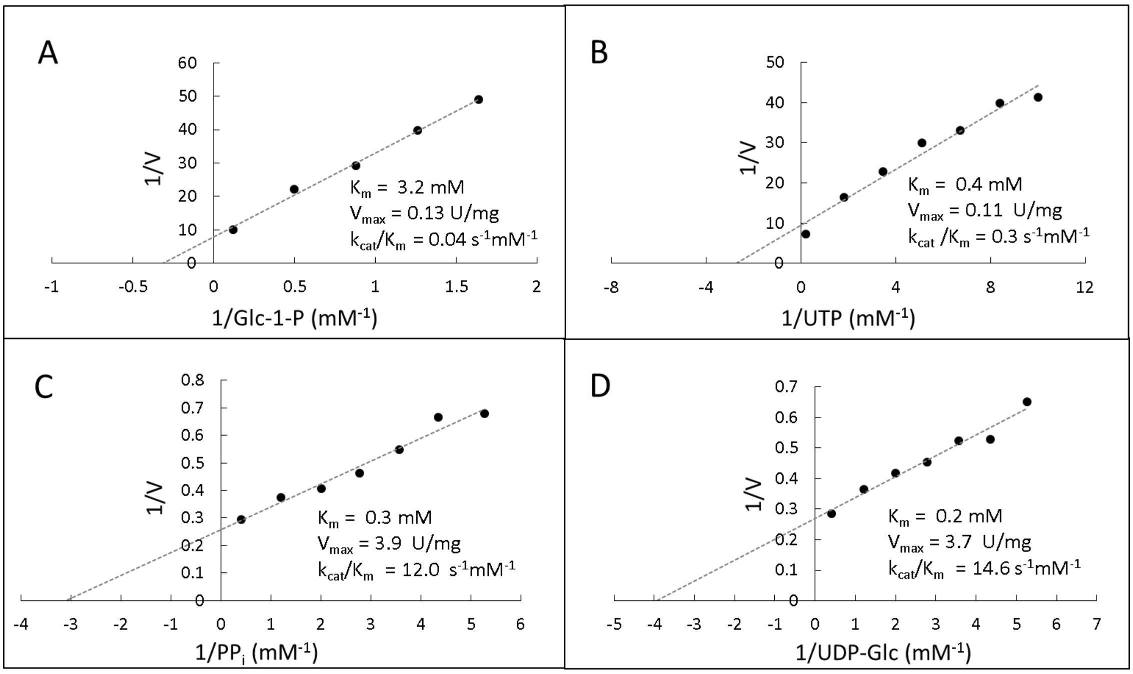

Fig. S5. K_m values of *Arabidopsis* UAGPase2 with (A) Glc-1-P and (B) UTP (forward reaction), and with (C) UDP-Glc and (D) PPi (reverse reaction). Concentrations of fixed substrates when the other substrate was varied were: 5 mM UTP; 10 mM Glc-1-P; 2.5 mM UDP-Glc; and 2.5 mM PPi. V, activity (units/mg protein). See Material and Methods for other details of the assays used.
