# Supplementary material for: Substrate Specificity and Inhibitor Sensitivity of Plant UDP-Sugar Producing Pyrophosphorylases
Source: Front Plant Sci. 2017 Sep 20;8:1610. doi: 10.3389/fpls.2017.01610 (PMC5609113; doi:10.3389/fpls.2017.01610)
Supplement: Supplementary file 8 [file Image_6.PDF]

A

	Activity (%)± SD
Glc-1-P	100 ± 1.2
Fru-2-P	27.0 ± 4.8
Fru-1-P	8.7 ± 2.4
Fru-6-P	NA
Fru-2,6-BP	NA

B

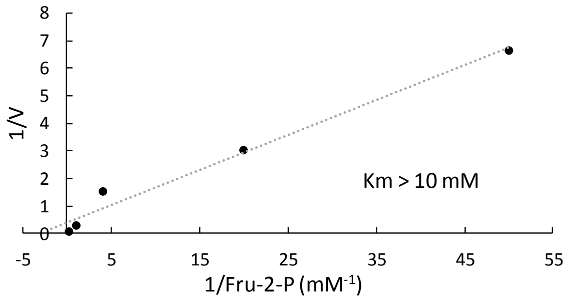

Fig. S6. Determination of barley UGPase activity with Glc-1-P and different fructose-phosphates (A) and determination of its K_m with Fru-2-P (B). In **(A)**, activities were assayed with 1 mM of a given sugar-phosphate and 1 mM UTP. SD, standard deviation from at least 3 experiments. **(B)** Fru-2-P was varied from 0.02 to 4 mM. All assays contained UTP at 1 mM. 100% activity refers to 1459 units per mg protein). V, activity (units/mg protein); NA, no activity.
