# Supplementary material for: Substrate Specificity and Inhibitor Sensitivity of Plant UDP-Sugar Producing Pyrophosphorylases
Source: Front Plant Sci. 2017 Sep 20;8:1610. doi: 10.3389/fpls.2017.01610 (PMC5609113; doi:10.3389/fpls.2017.01610)
Supplement: Supplementary file 9 [file Image_7.PDF]

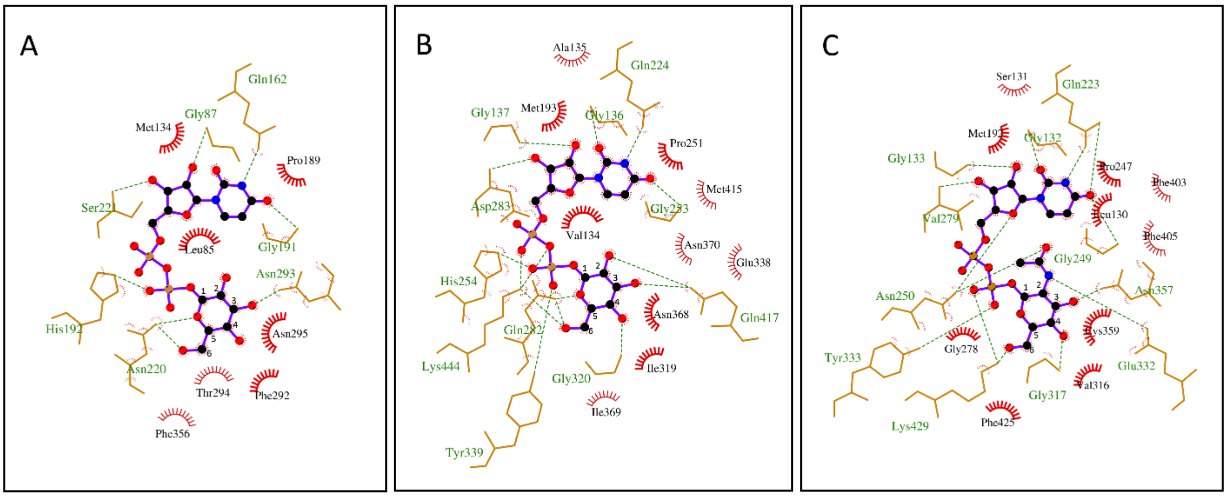

Fig. S7. Schematic representation of UDP-Glc binding to *Arabidopsis* UGPase1 (A) and *Arabidopsis* USPase (B), and of UDP-GlcNAc binding to *Arabidopsis* UAGPase2 (C). Names of aa which interact (or are in proximity) with the UDP-sugar are presented in black (putative hydrophobic interaction) or green font (hydrogen bond). Numbers for aa refer to actual aa sequence of a given enzyme. Active sites of the pyrophosphorylases were analyzed and presented using LigPlot+ , based on the resolved crystal structure of *At*UGP1 (2ICY) and homology models of *At*USPase and *At*UAGPase2, based on crystal structures of *Leishmania* USPase (PDB 3OH4) (Dickmanns et al., 2011) and human UAGPase (PDB 1JV1) (Peneff et al., 2001), respectively.
